# DNA barcoding: complementing morphological identification of mosquito species in Singapore

**DOI:** 10.1186/s13071-014-0569-4

**Published:** 2014-12-12

**Authors:** Abigail Chan, Lee-Pei Chiang, Hapuarachchige C Hapuarachchi, Cheong-Huat Tan, Sook-Cheng Pang, Ruth Lee, Kim-Sung Lee, Lee-Ching Ng, Sai-Gek Lam-Phua

**Affiliations:** Environmental Health Institute, National Environment Agency, 11 Biopolis Way, Helios Block, #06-05/08, Singapore, 138667 Singapore; Shizenature Pte Ltd, 3016, Eastech, Bedok North Avenue 4, #02-13, Singapore, 489947 Singapore; School of Life Sciences and Chemical Technology, Ngee Ann Polytechnic, 535, Clementi Road, Singapore, 599489 Singapore

**Keywords:** DNA barcode, COI, Identification, Taxonomy, Mosquitoes, Phylogeny

## Abstract

**Background:**

Taxonomy that utilizes morphological characteristics has been the gold standard method to identify mosquito species. However, morphological identification is challenging when the expertise is limited and external characters are damaged because of improper specimen handling. Therefore, we explored the applicability of mitochondrial cytochrome C oxidase subunit 1 (*COI*) gene-based DNA barcoding as an alternative tool to identify mosquito species. In the present study, we compared the morphological identification of mosquito specimens with their differentiation based on *COI* barcode, in order to establish a more reliable identification system for mosquito species found in Singapore.

**Methods:**

We analysed 128 adult mosquito specimens, belonging to 45 species of 13 genera. Phylogenetic trees were constructed for *Aedes, Anopheles, Culex* and other genera of mosquitoes and the distinctive clustering of different species was compared with their taxonomic identity.

**Results:**

The *COI*-based DNA barcoding achieved a 100% success rate in identifying the mosquito species. We also report *COI* barcode sequences of 16 mosquito species which were not available previously in sequence databases.

**Conclusions:**

Our study utilised for the first time DNA barcoding to identify mosquito species in Singapore. *COI*-based DNA barcoding is a useful tool to complement taxonomy-based identification of mosquito species.

**Electronic supplementary material:**

The online version of this article (doi:10.1186/s13071-014-0569-4) contains supplementary material, which is available to authorized users.

## Background

Morphological identification is the conventional, gold standard method to identify mosquito species based on their external characters. Different mosquito species exhibit distinguishable morphological features, which are utilized in taxonomic keys such as Bram [1], Harrison and Scanlon [2] and Rattanarithikul [3-7] to identify individual species. However, morphological identification requires experienced taxonomists and the method itself is highly time-consuming, especially in the hands of the less-experienced researchers. Moreover, incomplete identification is often encountered when important morphological features such as scales and bristles are damaged as a result of improper specimen handling. In addition, similar morphological characters shared by members of species complexes make identification a difficult task based on taxonomic keys alone [8]. Furthermore, most of the taxonomic keys are limited to adult female mosquitoes and fourth instar larvae because many of the morphological characteristics are not well developed in early larval stages. Moreover, many species of the *Culex* subgenus *Lophoceraomyia* are recognised on the basis of differences in male antenna, palpus, proboscis and genitalia, making the morphological identification of their female counterparts difficult [9]. These limitations restrict the applicability of existing taxonomic keys for the identification of certain mosquito species. Therefore, there is a need for an alternative, universally-applicable method to support the existing mosquito identification methods.

DNA barcoding is a molecular method that is becoming increasingly popular for the identification of animal species, based on partial mitochondrial DNA sequences [10,11]. This method is based on the concept that every species has a unique genetic identity [11,12]. A DNA barcode is a short standardised sequence of DNA that can be used as a genetic maker for species identification [11,13]. Early studies on DNA barcoding have used the nuclear internal transcribed spacer 2 [14], cytochrome b oxidase [15,16], 12S rRNA [17,18] and nicotinamide adenine dinucleotide dehydrogenase [19,20] as target genes. In recent years, however, the mitochondrial cytochrome c oxidase subunit 1 (*COI*) gene has gained increasing popularity, primarily because of the ease of using a universal set of primers to amplify the gene and its ability to provide a higher sequence variation at inter-species than at intra-species level [10]. *COI* gene-based DNA barcoding is, therefore, an alternative species identification method that can easily be standardized to obtain comparable results from different sources.

Herbert and co-workers [10,11] proposed using a 658 base pair (bp) region of the *COI* gene as a universal marker to barcode animal life. Previous studies have proven that *COI* gene is an efficient and useful barcode for the identification of metazoans, including mosquitoes [21-26]. However, *COI* barcode may not be universally applicable to identify all animal species. For example, *COI*-based barcoding has not been promising in identifying fungi and plant species [27-29]. Likewise, *COI* barcoding has failed to distinguish certain mosquito species of *Anopheles* and *Culex* [21-25]. Kumar et al., [23] reported that two closely related mosquito species of the genus *Ochlerotatus* could not be differentiated using their *COI* barcode. On the other hand, the barcoding approach has other common limitations as well. The recombination within mitochondrial genes may lead to complex sequence patterns when species with divergent mitochondrial DNA genomes interbreed, resulting in inconclusive identification. Moreover, the success of DNA barcoding is dependent on the availability of representative sequences for comparison. DNA barcoding approach fails if there are insufficient reference sequences in databases for comparison and analysis [8].

In instances where *COI* barcode fails to accurately identify certain mosquito species, a multi locus approach has been proposed [24]. By utilizing other gene markers and combining the datasets, the accuracy of identification can be increased. These observations, therefore, testify the need to use integrated datasets, including genomic, morphological and ecological data, to further understand the species diversity of the animal kingdom [30]. In the present study, we explored the applicability of *COI*-based DNA barcoding in the identification of mosquito species in Singapore. We compared the identification based on morphology and *COI*-based barcoding of 45 mosquito species belonging to 13 genera. By using both methods, we sought to establish a more reliable and standardized identification system for mosquito species found in Singapore.

## Methods

### Mosquito collection and identification

Mosquitoes were collected from various parts of Singapore from 2003 to 2012. Adult mosquito specimens were collected using BG-sentinel traps (BioGents AG, Germany), CO_2_ light traps, human baited net traps and human landing catch method. Larval samples were collected using the dipping method during field surveillance activities. Laboratory strains of several mosquito species, namely *Aedes* (*Stegomyia*) *aegypti* (Linneaus, 1762)*, Anopheles* (*Anopheles*) *sinensis* (Wiedemann, 1828)*, Culex* (*Culex*) *vishnui* (Theobald, 1901), *Culex* (*Culex*) *pseudovishnui* (Colless, 1957) *Culex* (*Culex*) *quinquefasciatus* (Say, 1823), *Lutzia* (*Metalutzia*) *fuscana* (Wiedermann, 1820) and *Culex (Culex) mimulus* (Edwards, 1915) that were colonised at the Environmental Health Institute (EHI) were also included in our analysis.

Field collected larvae were reared individually to adults. The imagos were identified by experienced taxonomists at EHI according to taxonomic keys [1-7,31-35]. A reference number was assigned to each adult mosquito which then was deposited as voucher specimens in the EHI mosquito repository.

### DNA extraction

In order to preserve voucher specimens for future references, DNA was extracted from the fore-, mid-, and hindlegs (n = 3) from one side of each mosquito. Legs were removed using clean, sterile forceps and were homogenised using a mixer mill (Retsch Mixer Mill MM301). Total DNA was extracted using the DNeasy blood and tissue kit (Qiagen, Hilden, Germany), according to manufacturer’s instructions. The extracted DNA was stored at -20°C until further analysis.

### Polymerase Chain Reaction and DNA sequencing

A 735 bp region flanking the mitochondrial *COI* gene was amplified by polymerase chain reaction (PCR) using following primers: forward 5’- GGATTTGGAAATTGATTAGTTCCTT - 3’ and reverse 5’ – AAAAATTTTAATTCCAGTTGGAACAGC – 3’ [23]. The 50 μl PCR reaction consisted of 5 μl of extracted DNA, 1.5 mM MgCl_2_ (Promega, USA), 0.2 mM dNTPs (Promega, USA), 1x reaction buffer (Promega, USA), 1.5 U Taq DNA polymerase (Promega, USA), and 0.3 μM of each primer.

PCR reaction conditions were as follows: an initial denaturation of 95°C for 5 minutes, followed by 5 cycles of denaturation at 94°C for 40 seconds, annealing at 45°C for 1 minute and extension at 72°C for 1 minute. The amplification reaction was continued for another 35 cycles of denaturation at 94°C for 40 seconds, annealing at 51°C for 1 minute and extension at 72°C for 1 minute followed by a final extension at 72°C for 10 minutes [23].

Amplicons were visualised on 1.5% agarose gels stained with GelRed (Biotium Inc., USA). PCR products were purified using the Purelink PCR purification kit (Invitrogen Corp., USA), according to manufacturer’s recommendations. Sequencing was performed at a commercial laboratory according to the recommended protocol for BigDye Terminator Cycle Sequencing kit (Applied Biosystems, USA).

*COI* sequences generated in this study were deposited in the GenBank (http://www.ncbi.nlm.nih.gov) and Barcode of Life (BOLD) (http://www.boldsystems.org) databases [NCBI: KF564650 to KF564674, KF564678 to KF564740, KF564643 to KF564778 and KM609455 to KM609458].

### Phylogenetic analyses and genetic distance calculation

Contiguous sequences of *COI* gene were generated from forward and reverse chromatograms using Lasergene 8.0 software suite (DNASTAR Inc., USA). Completed sequences were aligned using the Clustal W algorithm [36] implemented in BioEdit v7.0.5 software [37]. Separate phylogenetic trees were constructed by using the neighbour joining algorithm implemented in MEGA 6.06 software suite [38]. Parameters for phylogenetic construction included a Kimura-2 parameter substitution model with gamma distributed rates using the nearest neighbour interchange heuristic search method. Robustness of clustering was determined by bootstrap analysis with 1000 replicates. Reference DNA sequences were obtained from the GenBank and BOLD databases. The pairwise distance between individual species within *Aedes, Anopheles, Culex* and other genera described in this study was calculated using MEGA 6.06 software package [38].

### Ethical consideration

All specimens belonging to species of mosquitoes analysed in the present study were collected as part of the vector surveillance and control programmes conducted by Environmental Health Department of National Environment Agency and Singapore Armed Forces. EHI serves as the reference centre for the identification of field collected adult and larval specimens under the above programmes. The study was approved by the Project Evaluation Committee of EHI, National Environment Agency, Singapore (Reference No. TS058).

## Results and discussion

### Mosquito specimens and collection habitats

In total, 128 mosquito specimens belonging to 45 species of 13 genera were analysed. Of the 128 specimens, 83 mosquitoes (65%) were collected from rural habitats. Those habitats included forested areas such as Pulau Ubin, Sungei Buloh Wetland Reserve, Pulau Tekong and military training areas. Of the remaining, 32 specimens (25%) were from urban housing estates and private houses. The majority of urban specimens were collected as larvae and were reared into adults before identification. Colonised mosquitoes (n = 13) constituted 10% of the specimens. Information about the exact locations of specimen collection is given in Additional file 1.

### *COI*-based DNA barcoding accurately identified all species of *Aedes, Culex, Anopheles* and other genera of mosquitoes

Accurate identification of mosquito species is instrumental in vector control programmes because only a handful of mosquito species plays an important role in disease transmission [39]. Advancement in DNA-based molecular techniques allows us to complement the taxonomical identification of mosquito species. Our analysis included 10 species of *Aedes* and *Verrallina* (n = 30), 13 species of *Culex* and *Lutzia* (n = 42), nine species of *Anopheles* (n = 33) and 13 species of other genera (n = 23) of mosquitoes. Other genera included *Aedeomyia, Armigeres, Coquillettidia, Ficalbia, Mansonia, Toxorhynchites, Uranotaenia,* and *Zeugomyia*.

**Figure 1:**
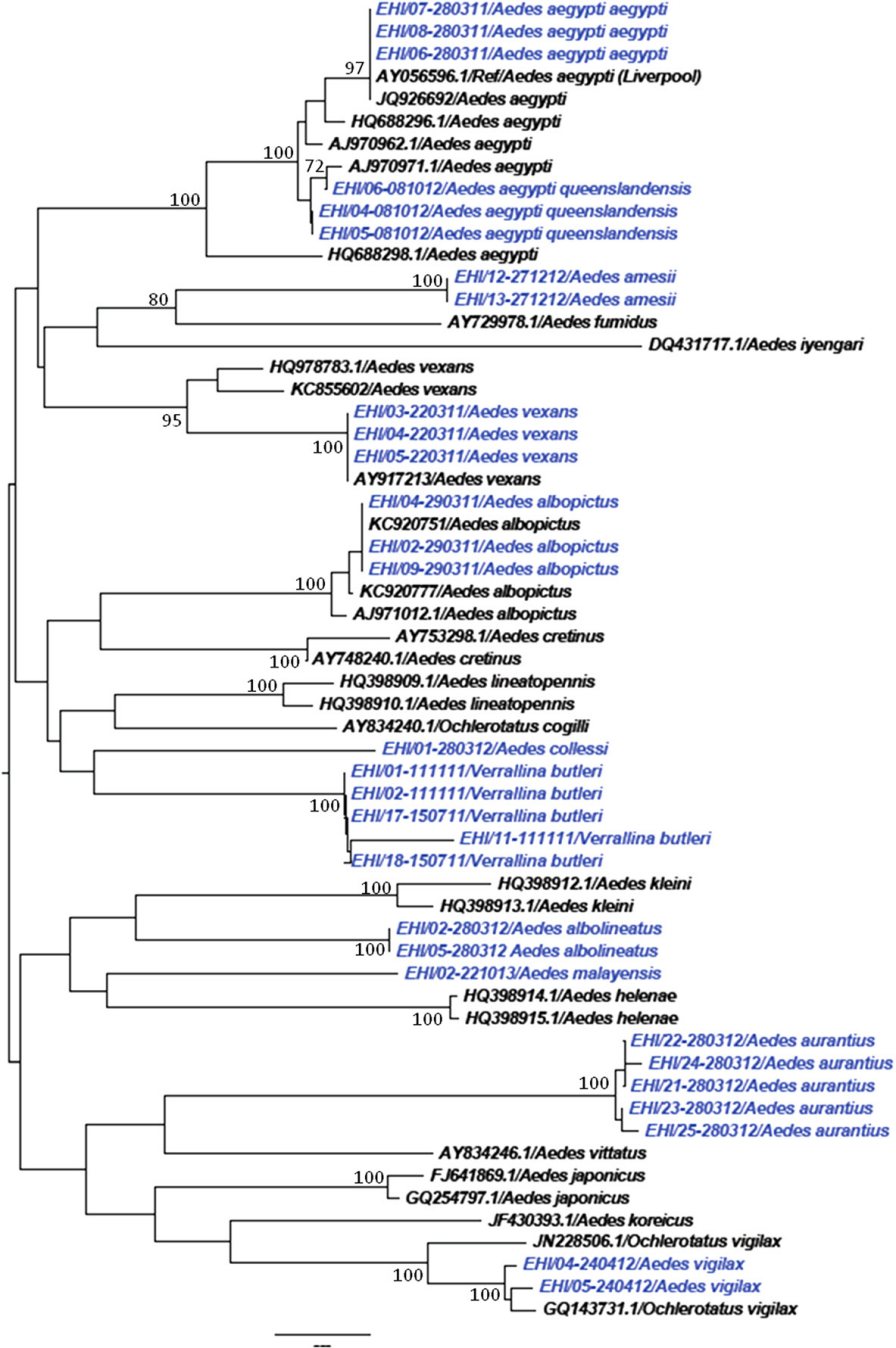
**Phylogenetic tree based on** ***COI*** **sequences of** ***Aedes*** **and** ***Verrallina*** **spp. mosquitoes.** An alignment of *COI* gene sequences (440 bp) was used to construct the neighbour joining tree in MEGA 6.06 software. Numbers displayed on branches are the bootstrap support obtained through 1000 replications. GenBank sequences are shown with accession numbers. Sequences starting with “EHI” were generated during this study and are highlighted in blue.

**Figure 2:**
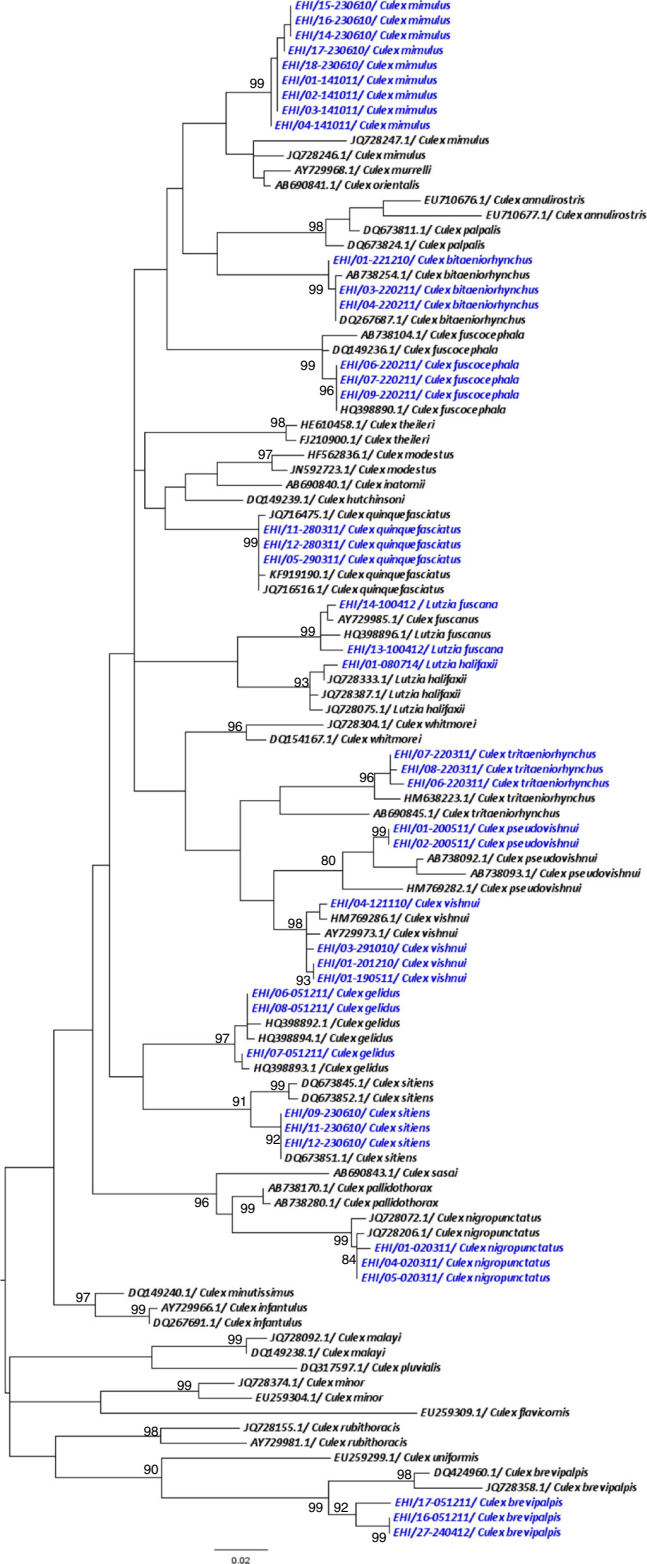
**Phylogenetic tree based on** ***COI*** **sequences of** ***Culex*** **and** ***Lutzia*** **spp. mosquitoes.** A 432 bp-region of the *COI* gene was used to construct the neighbour joining tree in MEGA 6.06 software. Numbers displayed on branches are the bootstrap support obtained through 1000 replications. GenBank sequences are shown with accession numbers. Sequences starting with “EHI” were generated during this study and are highlighted in blue.

**Figure 3:**
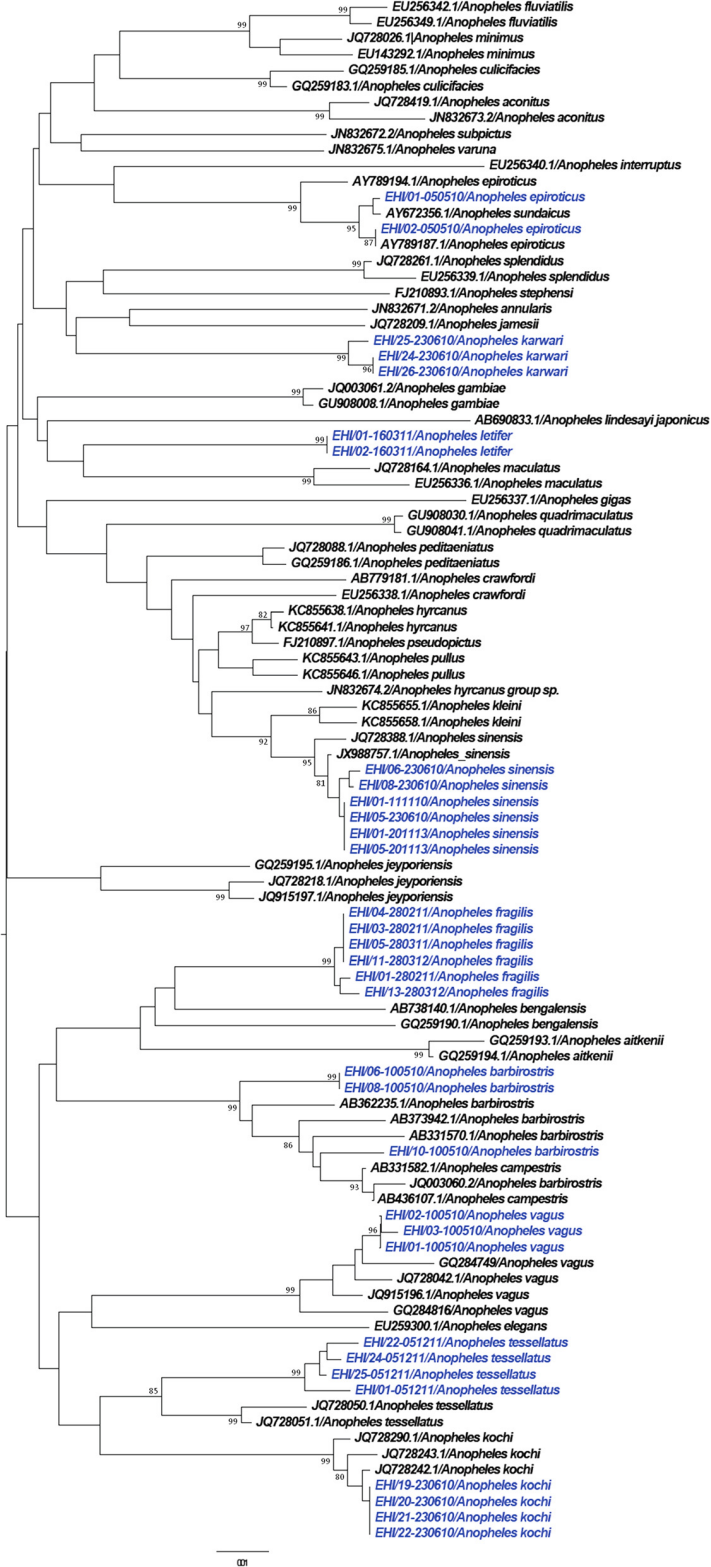
**Phylogenetic tree based on** ***COI*** **sequences of** ***Anopheles spp.*** **mosquitoes.** A 452 bp-region of the *COI* gene was used to construct the neighbour joining tree in MEGA 6.06 software. Numbers displayed on branches are the bootstrap support obtained through 1000 replications. GenBank sequences are shown with accession numbers. Sequences starting with “EHI” were generated during this study and are highlighted in blue.

**Figure 4:**
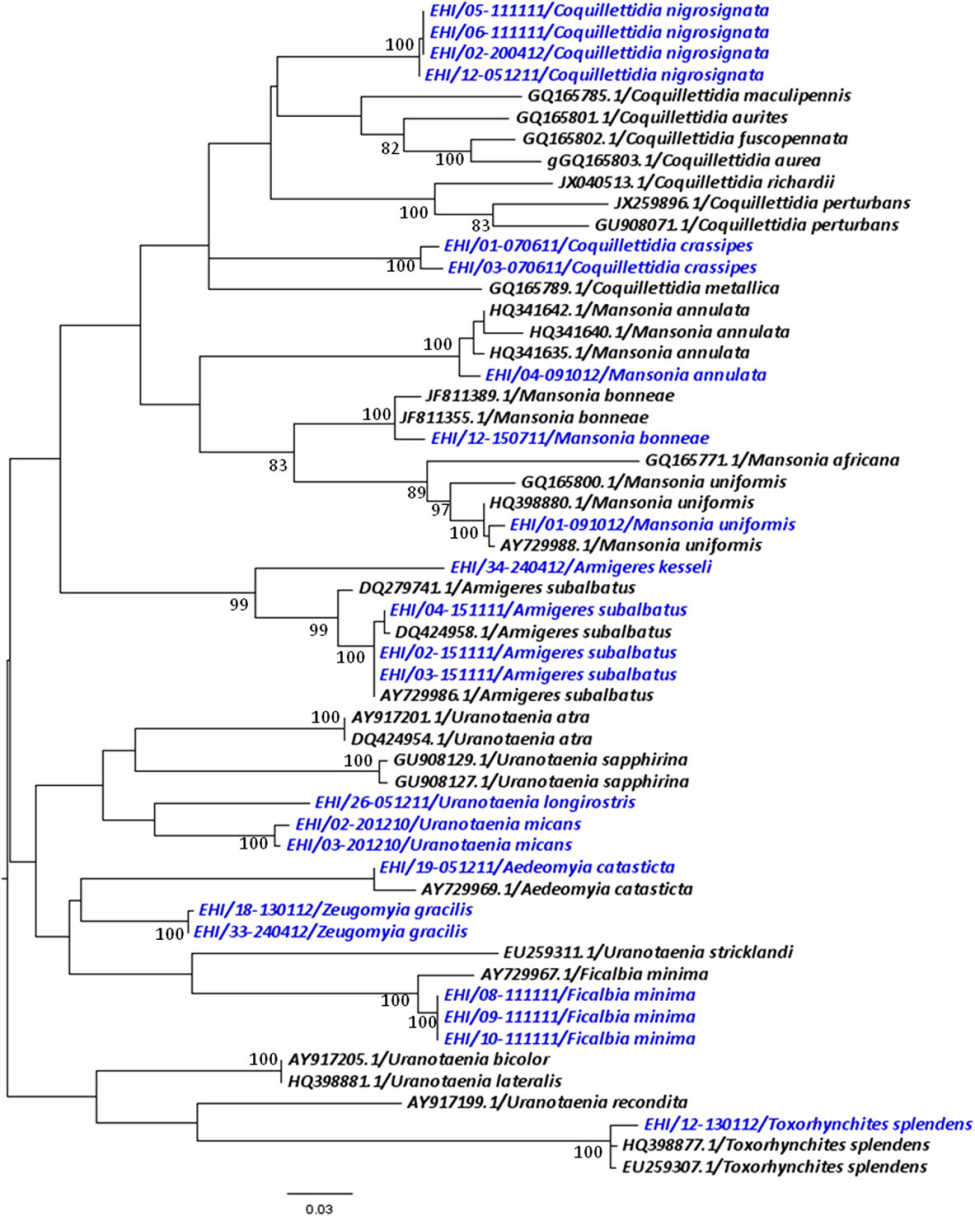
**Phylogenetic tree based on** ***COI*** **sequences of other genera of mosquitoes.** A 447 bp-region of the *COI* gene was used to construct the neighbour joining tree in MEGA 6.06 software. Numbers displayed on branches are the bootstrap support obtained through 1000 replications. GenBank sequences are shown with accession numbers. Sequences starting with “EHI” were generated during this study and are highlighted in blue.

As illustrated in Figures 1, 2, 3 and 4, *COI*-based phylogenetic analyses showed distinct clustering of individual species within each genus with strong bootstrap support. Clustering patterns agreed with the morphological identification, enabling the differentiation of individual species based on *COI* sequences. Pairwise distance analyses of *COI* sequences showed that inter-species “barcode gap” exceeded the proposed cut-off of 2-3% [30], supporting the ability of *COI* barcode to differentiate analysed species (Additional files 2, 3, 4 and 5). Furthermore, our sequences clustered with those of similar species from other endemic regions reported in the NCBI and BOLD databases. All species analysed in this study (n = 45) could, therefore, be identified based on their *COI* barcode, yielding a 100% compatibility between molecular and taxonomic identification, indicating that *COI* barcode is a useful tool to complement taxonomy for the identification of mosquito species.

### *COI* barcode differentiated morphologically similar species of *Aedes* and *Culex* genera

It was noteworthy that several morphologically similar *Aedes* and *Culex* species could be differentiated based on the *COI* barcode.

**Figure 5:**
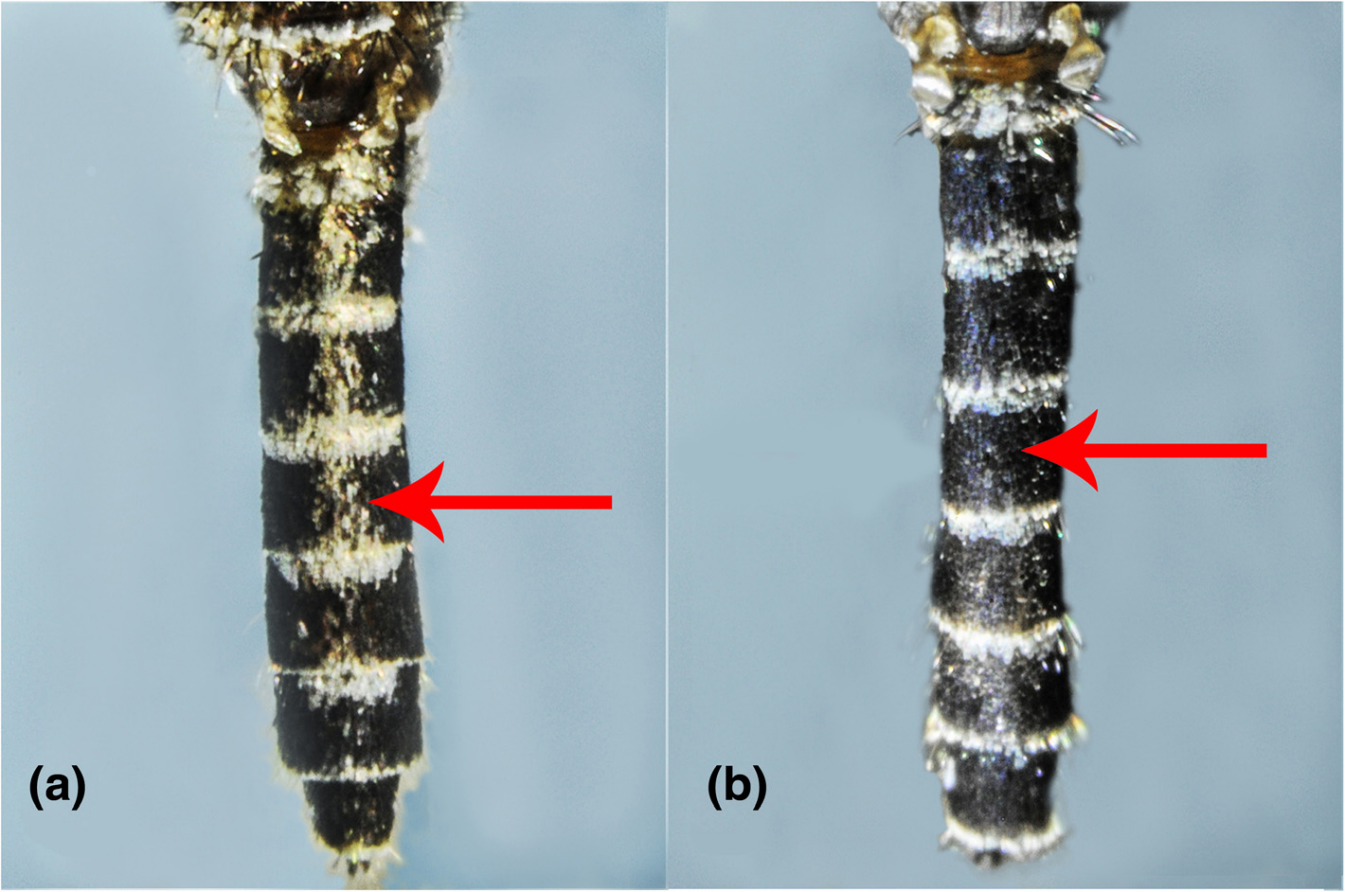
**Morphological comparison of the abdominal terga of** ***Ae. aegypti queenslandensis*** **and** ***Ae. aegypti aegypti.*** **(a)**: *Ae. aegypti queenslandensis:* unbroken median stripes of pale scales along the abdominal terga. **(b)**: *Ae. aegypti aegypti:* no median stripes of pale scales from terga II to VII.

One such pair was *Aedes* (*Stegomyia*) *aegypti aegypti* and *Aedes* (*Stegomyia*) *aegypti queenslandensis* (Theobald, 1901). Adult stages of these two forms are differentiated based on white scales on the abdominal terga. In *Ae. aegypti queenslandensis*, there are pale scales that appear as unbroken median stripes on the abdominal terga II to VII (Figure 5a). These pale scales are not seen on the abdominal terga of *Ae. aegypti aegypti* (Figure 5b) [33]. Given such minute differences, it is difficult to differentiate these two forms when scales on the abdominal terga are rubbed off. However, as illustrated in Figure 1, *COI* barcode-based phylogeny differentiated *Ae. aegypti queenslandensis* from *Ae. aegypti aegypti.* According to *COI* sequence analysis, the genetic distance between *Ae. aegypti aegypti and Ae. aegypti queenslandensis* varied from 1.5% to 1.9% (Additional file 2)*.* This distance was lower than the “barcode gap” proposed to differentiate vertebrate (2%) and invertebrate (3%) species based on *COI* sequences [30]. Therefore, *Ae. aegypti aegypti* and *Ae. aegypti queenslandensis* were not considered as two subspecies. This observation agreed with previous descriptions of *Ae. aegypti* as a group of highly polymorphic mosquitoes. Mattingly described four morphological forms of *Ae. aegypti* which were distinguishable based on colour characters; a pale form (*Ae. aegypti queenslandensis*), an intermediate (type) form (*Ae. aegypti aegypti*), a dark form (*Ae. aegypti formosus*) and a fourth form (*Ae. aegypti mascarensis*) [40,41]. Based on morphology and habitat differences, Mattingly concluded that both *Ae. aegypti formosus* and *Ae. aegypti mascarensis* were clearly subspecies, but classified *Ae. aegypti queenslandensis* as a variety (var.) [41]*.* According to previous descriptions, *Ae. aegypti aegypti* and *Ae. aegypti queenslandensis* share a similar geographical distribution pattern [41]. It is known that the presence of subspecies as well as their geographic and ecological separation may affect the potential of a mosquito species to act as a vector. *Aedes aegypti* is considered as the primary vector of Dengue virus (DENV) in endemic regions, including Singapore [42]. Even though preliminary findings of a previous study have shown no significant differences in oral infection of DENV-2 between pale (*Ae. aegypti queenslandensis*) and dark (*Ae. aegypti aegypti*) forms of *Ae. aegypti* in Thailand [43], no extensive studies have so far been carried out to determine the differences in vectorial capacity between *Ae. aegypti queenslandensis* and *Ae. aegypti aegypti.* In this context, the ability of *COI* barcoding to differentiate *Ae. aegypti queenslandensis* and *Ae. aegypti aegypti* is especially advantageous.

**Figure 6:**
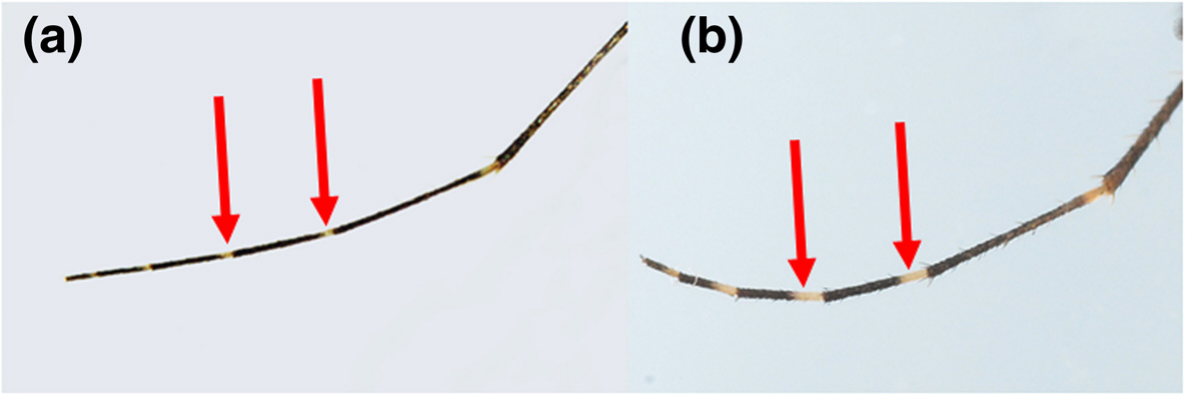
**Morphological comparison of the hind tarsa of** ***Ae. vexans*** **and** ***Ae. vigilax*** **. (a)**: *Ae. vexans:* pale basal bands in less than a quarter of the length of hind tarsomeres. **(b)**: *Ae. vigilax:* pale basal bands covering more than a quarter of the length of hind tarsomeres.

**Figure 7:**
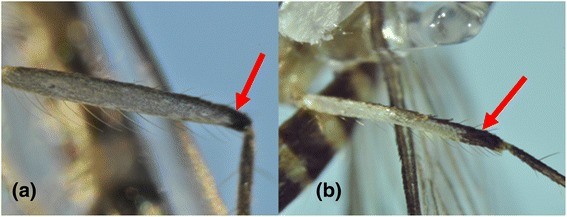
**Morphological comparison of the hind femur of** ***Cx. vishnui*** **and** ***Cx. pseudovishnui.*** **(a)**: *Cx. vishnui*: hind femur with apical dark band not well contrasted with pale scales on the hind femur. **(b)**: *Cx. pseudovishnui*: hind femur with apical dark band well contrasted with pale scales on the hind femur.

Likewise, *Aedes* (*Aedimorphus*) *vexans* (Meigen, 1830*)* and *Aedes* (*Ochlerotatus*) *vigilax* (Skuse, 1889*)* share similar morphological features such as pale scaling on the basal underside of proboscis, dark and white scales along the costa and subcosta of wings, and a narrow abdominal segment VIII which is nearly retracted into segment VII [34]. *Ae. vexans* has narrow basal pale bands that span over less than 1/4 of the length of hind tarsomeres (Figure 6a) [6]. On the other hand, *Ae. vigilax* has broad basal pale bands covering more than 1/4 of the length of hind tarsomeres (Figure 6b) [6]. Similarly, the taxonomical differentiation of *Cx. vishnui* and *Cx. pseudovishnui* also relies on subtle differences in the hind femur. *Cx. vishnui* has an apical dark band on the anterior surface of hind femur which is not well contrasted due to the presence of pale scales (Figure 7a), whereas it is well-contrasted in *Cx. pseudovishnui* (Figure 7b) [5]. Such subtle morphological differences pose a difficulty in accurate morphological identification of those species, especially in the hands of non-experienced taxonomists. Our results showed that *COI* barcode is able to differentiate those morphologically similar species (Figures 1 and 2) and thus complements their taxonomic identification.

**Figure 8:**
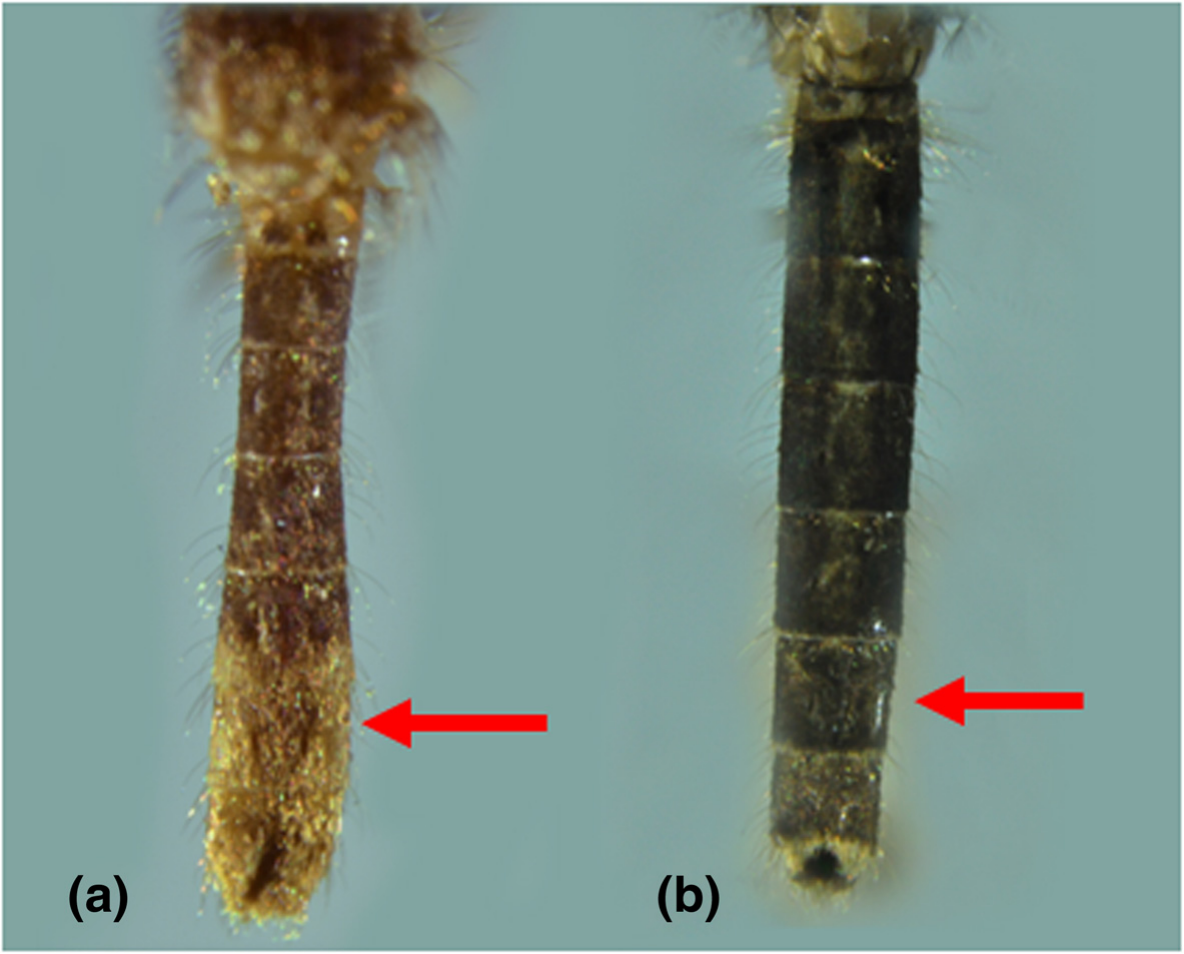
**Morphological comparison of the abdominal terga of** ***Lt. fuscana*** **and** ***Lt. halifaxii.*** **(a)**: *Lt. fuscana*: yellowish scales on abdominal terga. **(b)**: *Lt. halifaxii*: entirely dark abdominal terga*.*

**Figure 9:**
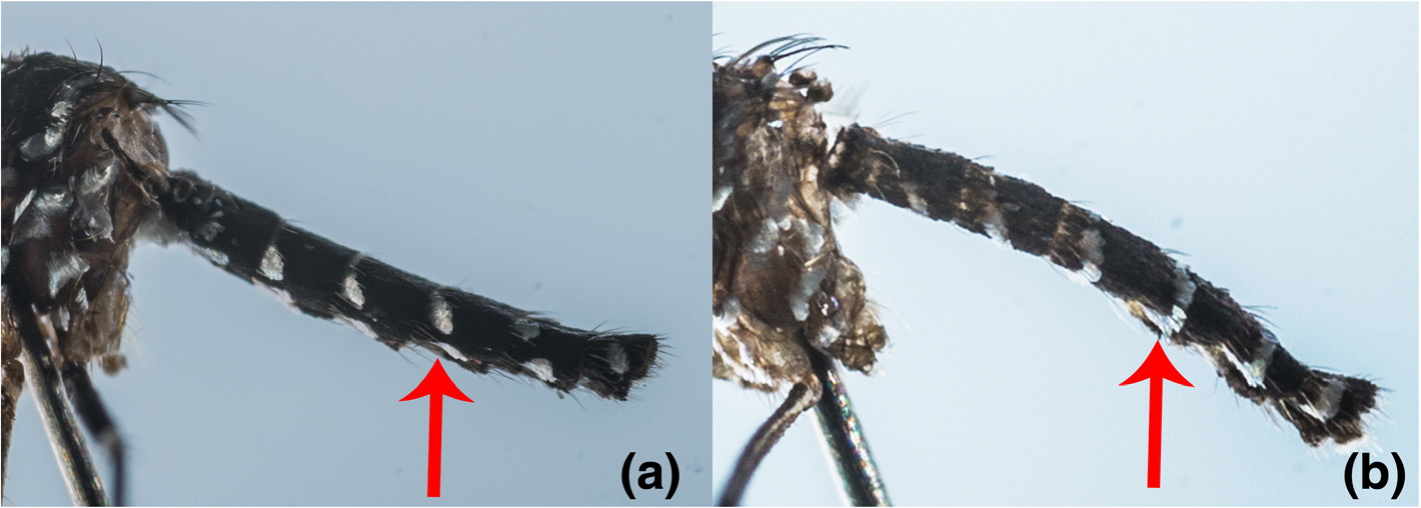
**Morphological comparison of the abdominal terga of** ***Ae. albopictus*** **and** ***Ae. malayensis*** **. (a)**: *Ae. albopictus*: dorsal white bands separated from the lateral spots on abdominal terga. **(b)**: *Ae. malayensis*: dorsal white bands connected to the lateral pale patches on abdominal terga*.*

**Figure 10:**
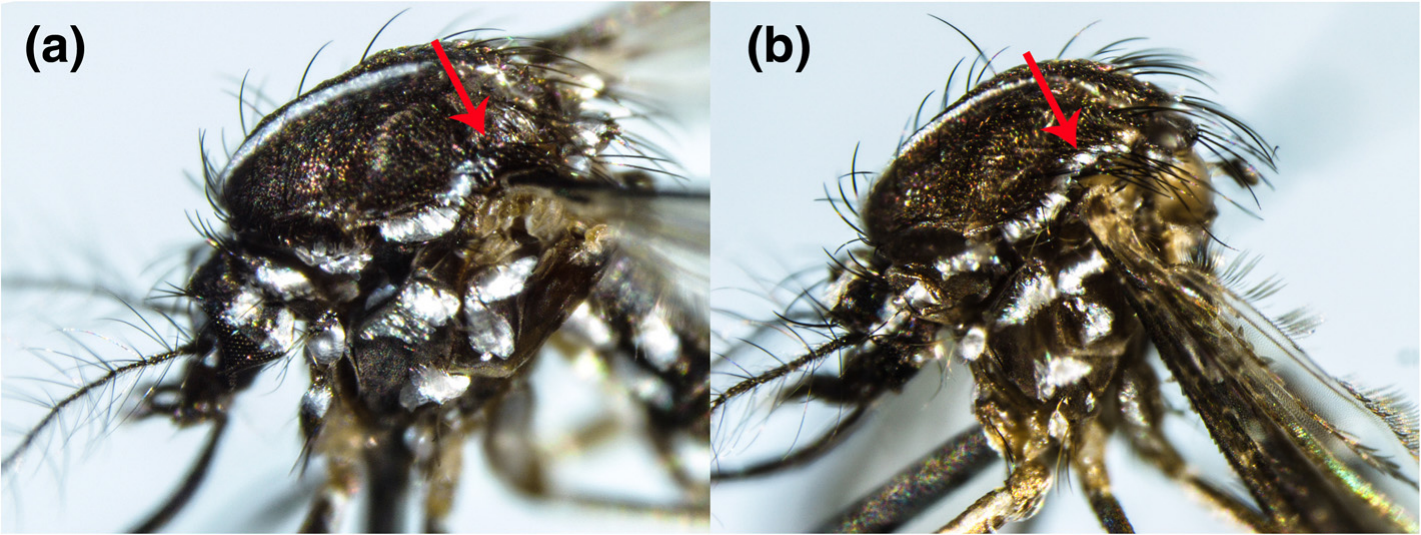
**Morphological comparison of the thorax of** ***Ae. albopictus*** **and** ***Ae. malayensis.*** **(a)**: *Ae. albopictus:* pale patches of scales do not extended towards the scutellum on the thorax. **(b)**: *Ae. malayensis*: pale scales extended towards the scutellum on the thorax.

Moreover, morphological identification of mosquitoes becomes a challenge when the morphological characterisation is stage-specific. For example, even though the adult mosquitoes of *Lt. fuscana* and *Lutzia* (*Metalutzia*) *halifaxii* (Theobald, 1903) can be distinguished based on differences of their abdominal terga (Figures 8a, b), their larval stages cannot be distinguished from each other [1,5]. Another example is *Aedes* (*Stegomyia*) *albopictus* (Skuse, 1894) and *Aedes* (*Stegomyia*) *malayensis* (Colless, 1962). Even though the adult mosquitoes can easily be distinguished based on differences of their abdominal terga area (Figures 9a, b) and supraalar (Figures 10a, b) [6], their larval stages are morphologically similar. As *Ae. albopictus* and *Ae. malayensis* coexist in the same habitats [32], larvae of both species are often found mixed during larval surveillance activities. Accurate identification of these two species is important as only *Ae. albopictus* is a vector of human pathogens such as DENV and Chikungunya virus. In contrast, adults of *Cx. vishnui* and *Cx. pseudovishnui* share many similar external characteristics (Figures 7a, b), which make them difficult to separate from each other. However, they are distinguishable at the larval stage. Nonetheless, rearing of larvae into adults as a requirement of taxonomic differentiation of certain species is time and resource intensive. Our findings showed that *COI* barcoding could differentiate these mosquito species (Figures 1 and 2) and thus be utilized to overcome such challenging scenarios. Therefore, complementing morphological identification with molecular characterisation has the potential to enhance vector surveillance capacity.

### DNA barcoding enhanced the identification of *Anopheles sinensis*

**Figure 11:**
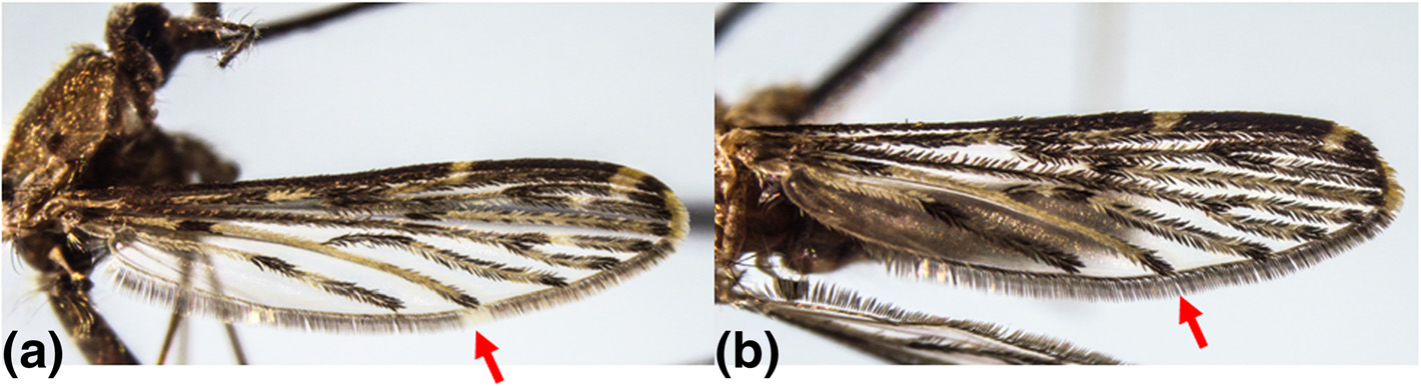
**Morphological comparison of the vein CuA of** ***An. sinensis.*** **(a)**: Pale fringe spot at vein CuA. **(b)**: Dark fringe spot at vein CuA.

An interesting phenomenon was observed when field-caught *An. sinensis* mosquitoes were subsequently colonised in the laboratory. According to taxonomic keys, *An. sinensis* is identified based on morphological characteristics such as apical pale bands on hind tarsomeres and the wing venation [2,4,31]. We observed differences in the wing venation of field-caught and laboratory colonised *An. sinensis* adults in Singapore. Reid [31] previously reported that approximately 75% of *An. sinensis* specimens had pale fringe spots at vein CuA. However, as shown in Figures 11a and b, our specimens of *An. sinensis* had both pale and dark fringe spots at the end of vein CuA. Nevertheless, all *COI* barcode sequences of *An. sinensis* specimens clustered together in the phylogenetic tree (Figure 3), regardless of their morphological differences at the end of vein CuA. Therefore, despite a polymorphic nature of wing venation that may impede taxonomic identification, *COI* barcoding enabled us to confirm that those specimens were indeed *An. sinensis*. These observations also indicated that existing taxonomic keys for the identification of *An. sinensis* need to be revised further.

In summary, our findings suggested that *COI*-based DNA barcode can effectively be used when morphological traits of certain species do not clearly distinguish one species from another [44]. DNA barcodes also allow taxonomists to re-confirm the reference voucher specimens. For instance, the *COI* barcode of a voucher specimen initially identified as *Culex* (*Culex*) *bitaeniorhynchus* (Giles. 1901) in our samples clustered with *Cx. vishnui* in the phylogenetic analysis*.* This prompted us to re-visit the voucher specimen which was then correctly re-identified as *Cx. vishnui* after a thorough morphological analysis. Therefore, *COI*-based molecular characterisation has immense potential to be used as a complementary tool for the identification of mosquito species.

## Conclusions

DNA barcoding is a useful tool to complement taxonomy for the identification of mosquito species. In the present study, we demonstrated that mitochondrial *COI* gene-based DNA barcoding was comparable to morphological identification for the differentiation of 45 mosquito species analysed. In our analyses, *COI* barcode was even able to differentiate several mosquito species that were difficult to distinguish morphologically. However, empirical evidence has shown that *COI*-based barcoding is not successful all the time [21-23]. Firstly, the limited availability of sequences to be used as references for comparison has restricted its usage on species identification. In the present study, we provided *COI* gene sequences of 16 mosquito species which were not available previously in sequence databases (Additional file 1). We believe that those new sequences would contribute to the on-going global effort to standardise DNA barcoding as a molecular means of species identification by the Consortium for the Barcode of Life (CBOL) [8]. Secondly, the cut-off limit of “barcode gap” for species differentiation still remains controversial. Both of these limitations have implications on the identification of new species based on barcoding alone. Therefore, *COI*-based DNA barcoding may not always be useful on its own, but would rather be an alternative tool to complement morphological identification. The use of integrated datasets and multi locus analyses will further enhance the molecular identification. Although the taxonomic keys have been developed to identify different genera of mosquitoes from various geographical settings, little progress has been made to classify mosquito species based on phylogenetic relationships [45,46]. The phylogeny-assisted DNA barcode analyses enable us to refine the taxonomic identification and further understand the genetics and evolution of mosquito species in endemic habitats.

## Additional files

Additional file 1:
**Location of specimen collection and sequence accession information of mosquito specimens (n = 128) included in the study.**
Additional file 2:
**Pairwise nucleotide distance of mitochondrial cytochrome C oxidase subunit 1 (** ***COI*** **) gene of** ***Aedes and Verrallina*** **species.**
Additional file 3:
**Pairwise nucleotide distance of mitochondrial cytochrome C oxidase subunit 1 (** ***COI*** **) gene of** ***Anopheles*** **species.**
Additional file 4:
**Pairwise nucleotide distance of mitochondrial cytochrome C oxidase subunit 1 (** ***COI*** **) gene of** ***Culex*** **and** ***Lutzia*** **species.**
Additional file 5:
**Pairwise nucleotide distance of mitochondrial cytochrome C oxidase subunit 1 (** ***COI*** **) gene of species belonging to other genera.**

## Abbreviations

BOLD: Barcode of Life
CBOL: Consortium for the Barcode of Life
COI: Cytochrome c oxidase subunit 1
DENV: Dengue virus
EHI: Environmental Health Institute
NCBI: National Centre for Biotechnology Information
PCR: Polymerase chain reaction
