# Supplementary material for: DNA barcoding: complementing morphological identification of mosquito species in Singapore
Source: Parasit Vectors. 2014 Dec 12;7:569. doi: 10.1186/s13071-014-0569-4 (PMC4282734; doi:10.1186/s13071-014-0569-4)
Supplement: Additional file 1: — Location of specimen collection and sequence accession information of mosquito specimens (n = 128) included in the study. [file 13071_2014_569_MOESM1_ESM.docx]

**Additional file 1. Location of specimen collection and sequence accession information of mosquito specimens (n=128) included in the study**

| **S/N** | **Species^§^** | **EHI repository ID** | **Genbank accession no./BOLD no.** | **Location** | **Latitude** | **Longitude** |
| --- | --- | --- | --- | --- | --- | --- |
| 1 | *Aedes (Stegomyia) aegypti* (Linnaeus 1762) *aegypti* | 06-280311 | KF564713/ SIN001-14 | Marsiling Drive | 1.4412 | 103.7760 |
| 2 | *Ae. aegypti aegypti* | 07-280311 | KF564651/ SIN002-14 | Marsiling Drive | 1.4412 | 103.7760 |
| 3 | *Ae. aegypti aegypti* | 08-280311 | KF564652/ SIN003-14 | Marsiling Drive | 1.4412 | 103.7760 |
| **4** | ***Aedes (Stegomyia) aegypti* (Linnaeus 1762) *queenslandensis* (Theobald 1901)** | **05-081012** | **KF564671/ SIN004-14** | **EHI laboratory strain** | **NA** | **NA** |
| **5** | ***Ae. aegypti queenslandensis*** | **04-081012** | **KF564670/ SIN005-14** | **EHI laboratory strain** | **NA** | **NA** |
| **6** | ***Ae. aegypti queenslandensis*** | **06-081012** | **KF564672/ SIN006-14** | **EHI laboratory strain** | **NA** | **NA** |
| **7** | ***Verrallina butleri* (Theobald 1901)** | **01-111111** | **KF564665/ SIN007-14** | **Sungei Buloh Wetland Reserve** | **1.4468** | **103.7301** |
| **8** | ***Ve. butleri*** | **02-111111** | **KF564666/ SIN008-14** | **Military training areas** | **NA** | **NA** |
| **9** | ***Ve. butleri*** | **11-111111** | **KF564667/ SIN009-14** | **Military training areas** | **NA** | **NA** |
| **10** | ***Ve. butleri*** | **17-150711** | **KF564668/ SIN010-14** | **Murai Farmway** | **1.3905** | **103.6960** |
| **11** | ***Ve. butleri*** | **18-150711** | **KF564669/ SIN011-14** | **Military training areas** | **NA** | **NA** |
| 12 | *Aedes (Stegomyia) albopictus* (Skuse 1895) | 09-290311 | KF564662/ SIN012-14 | Wall Street | 1.2834 | 103.8471 |
| 13 | *Ae. (Stegomyia) albopictus* | 04-290311 | KF564664/ SIN013-14 | Mcnair Road | 1.3209 | 103.8588 |
| 14 | *Ae. (Stegomyia) albopictus* | 02-290311 | KF564663/ SIN014-14 | Mount Pleasant Road | 1.3329 | 103.8304 |
| **15** | ***Aedes (Paraedes) collessi* (Mattingly 1958)** | **01-280312** | **KF564658/ SIN015-14** | **Murai Farmway** | **1.3905** | **103.6960** |
| 16 | *Ae. (Aedimorphus) vexans* | 03-220311 | KF564659/ SIN016-14 | Pulau Tekong | 1.4143 | 104.0387 |
| 17 | *Ae. (Aedimorphus) vexans* | 04-220311 | KF564660/ SIN017-14 | Pulau Tekong | 1.4143 | 104.0387 |
| 18 | *Ae. (Aedimorphus) vexans* | 05-220311 | KF564661/  SIN018-14 | Pulau Tekong | 1.4143 | 104.0387 |
| 19 | *Aedes (Ochlerotatus) vigilax* (Skuse 1889) | 04-240412 | KF564673/ SIN019-14 | Pulau Tekong | 1.4143 | 104.0387 |
| 20 | *Ae. (Ochlerotatus) vigilax* | 05-240412 | KF564674/ SIN020-14 | Pulau Tekong | 1.4143 | 104.0387 |
| **21** | ***Aedes (Mucidus) aurantius* (Theobald 1907)** | **23-280312** | **KF564653/ SIN021-14** | **Pulau Tekong** | **1.4143** | **104.0387** |
| **22** | ***Ae. (Mucidus) aurantius*** | **25-280312** | **KF564657/ SIN022-14** | **Pulau Tekong** | **1.4143** | **104.0387** |
| **23** | ***Ae. (Mucidus) aurantius*** | **22-280312** | **KF564655/ SIN023-14** | **Pulau Tekong** | **1.4143** | **104.0387** |
| **24** | ***Ae. (Mucidus) aurantius*** | **21-280312** | **KF564654/ SIN024-14** | **Pulau Tekong** | **1.4143** | **104.0387** |
| **25** | ***Ae. (Mucidus) aurantius*** | **24-280312** | **KF564656/ SIN025-14** | **Pulau Tekong** | **1.4143** | **104.0387** |
| **26** | ***Aedes (Stegomyia) malayensis* Colless 1962** | **02-221013** | **KM609455/ SIN129-14** | **Netheravon Road** | **1.3909** | **103.9803** |
| **27** | ***Aedes (Scutomyia) albolineatus* (Theobald 1904)** | **02-280312** | **KF564650/ SIN026-14** | **Pulau Tekong** | **1.4143** | **104.0387** |
| **28** | ***Ae. (Scutomyia) albolineatus*** | **05-280312** | **KF564678/ SIN027-14** | **Pulau Tekong** | **1.4143** | **104.0387** |
| **29** | ***Aedes (Lorrainea) amesii* (Ludlow 1903)** | **12-271212** | **KF564679/ SIN028-14** | **Pulau Tekong** | **1.4143** | **104.0387** |
| **30** | ***Ae. (Lorrainea) amesii*** | **13-271212** | **KF564680/ SIN029-14** | **Pulau Tekong** | **1.4143** | **104.0387** |
| 31 | *Anopheles (Anopheles) sinensis* Wiedemann 1828 | 01-111110 | KF564704/ SIN030-14 | Military training areas | NA | NA |
| 32 | *An. (Anopheles) sinensis* | 08-230610 | KF564686/ SIN031-14 | Yishun Ave 1 | 1.4220 | 103.8640 |
| 33 | *An. (Anopheles) sinensis* | 06-230610 | KF564685/ SIN032-14 | Woodlands | 1.3521 | 103.8198 |
| 34 | *An. (Anopheles) sinensis* | 05-230610 | KF564684/ SIN033-14 | Woodlands Road | 1.4201 | 103.7549 |
| 35 | *An. (Anopheles) sinensis* | 01-201113 | KM609456  SIN034-14 | EHI laboratory strain | NA | NA |
| 36 | *An. (Anopheles) sinensis* | 02-201113 | KM609457  SIN035-14 | EHI laboratory strain | NA | NA |
| **37** | ***Anopheles (Anopheles) fragilis* (Theobald 1903)** | **01-280211** | **KF564700/ SIN036-14** | **Woodlands** | **1.3521** | **103.8198** |
| **38** | ***An. (Anopheles) fragilis*** | **13-280312** | **KF564702/ SIN037-14** | **Woodlands** | **1.3521** | **103.8198** |
| **39** | ***An. (Anopheles) fragilis*** | **03-280211** | **KF564689/ SIN038-14** | **Woodlands** | **1.3521** | **103.8198** |
| **40** | ***An. (Anopheles) fragilis*** | **04-280211** | **KF564690/ SIN039-14** | **Woodlands** | **1.3521** | **103.8198** |
| **41** | ***An. (Anopheles) fragilis*** | **11-280312** | **KF564701/ SIN040-14** | **Woodlands** | **1.3521** | **103.8198** |
| **42** | ***An. (Anopheles) fragilis*** | **05-280311** | **KF564703/ SIN041-14** | **Woodlands** | **1.3521** | **103.8198** |
| 43 | *Anopheles (Cellia) tessallatus* Theobald 1901 | 25-051211 | KF564699/ SIN042-14 | Murai Farmway | 1.3905 | 103.6960 |
| 44 | *An. (Cellia) tessallatus* | 24-051211 | KF564698/ SIN043-14 | Murai Farmway | 1.3905 | 103.6960 |
| 45 | *An. (Cellia) tessallatus* | 01-051211 | KF564696/ SIN044-14 | Murai Farmway | 1.3905 | 103.6960 |
| 46 | *An. (Cellia) tessallatus* | 22-051211 | KF564697/ SIN045-14 | Murai Farmway | 1.3905 | 103.6960 |
| 47 | *Anopheles (Cellia) kochi* Dönitz 1901 | 21-230610 | KF564707/ SIN046-14 | Woodlands | 1.3521 | 103.8198 |
| 48 | *An. (Cellia) kochi* | 22-230610 | KF564708/ SIN047-14 | Woodlands | 1.3521 | 103.8198 |
| 49 | *An. (Cellia) kochi* | 19-230610 | KF564705/ SIN048-14 | Woodlands | 1.3521 | 103.8198 |
| 50 | *An. (Cellia) kochi* | 20-230610 | KF564706/ SIN049-14 | Bangkit Road | 1.3787 | 103.7734 |
| 51 | *Anopheles (Anopheles) barbirostris* Van der Wulp 1884 | 10-100510 | KF564681/ SIN050-14 | Sungei Buloh Wetland Reserve | 1.4467 | 103.7301 |
| 52 | *An. (Anopheles) barbirostris* | 06-100510 | KF564682/ SIN051-14 | Woodlands | 1.3521 | 103.8198 |
| 53 | *An. (Anopheles) barbirostris* | 08-100510 | KF564683/ SIN052-14 | Sungei Buloh Wetland Reserve | 1.4467 | 103.7301 |
| 54 | *Anopheles (Cellia) epiroticus* Linton & Harbach 2005 | 02-050510 | KF564688/ SIN053-14 | Lim Chu Kang Lane 9 | 1.4455 | 103.7101 |
| 55 | *An. (Cellia) epiroticus* | 01-050510 | KF564687/ SIN054-14 | Lim Chu Kang Lane 9 | 1.4455 | 103.7101 |
| 56 | *Anopheles (Cellia) vagus* Dönitz 1902 | 01-100510 | KF564691/ SIN055-14 | Lim Chu Kang Lane 6 | 1.4353 | 103.7134 |
| 57 | *An. (Cellia) vagus* | 02-100510 | KF564692/ SIN056-14 | Lim Chu Kang Lane 6 | 1.4353 | 103.7134 |
| 58 | *An. (Cellia) vagus* | 03-100510 | KF564693/ SIN057-14 | Lim Chu Kang Lane 6 | 1.4353 | 103.7134 |
| **59** | ***Anopheles (Anopheles) letifer* Sandosham 1944** | **01-160311** | **KF564694/ SIN058-14** | **Dairy Farm Road** | **1.3647** | **103.7733** |
| **60** | ***An. (Anopheles) letifer*** | **02-160311** | **KF564695/ SIN059-14** | **Rifle Range Road** | **1.3545** | **103.7952** |
| **61** | ***Anopheles (Cellia) karwari* (James 1902)** | **25-230610** | **KF564710/ SIN060-14** | **Chestnut Avenue** | **1.3708** | **103.7768** |
| **62** | ***An. (Cellia) karwari*** | **24-230610** | **KF564709/ SIN061-14** | **Chestnut Avenue** | **1.3708** | **103.7768** |
| **63** | ***An. (Cellia) karwari*** | **26-230610** | **KF564711/ SIN062-14** | **Chestnut Avenue** | **1.3708** | **103.7768** |
| 64 | *Culex (Culex) vishnui* Theobald 1901 | 01-201210 | KF564734/ SIN063-14 | Lor Chencharu | 1.4130 | 103.8296 |
| 65 | *Cx. (Culex) vishnui* | 01-190511 | KF564735/ SIN064-14 | EHI laboratory strain | NA | NA |
| 66 | *Cx. (Culex) vishnui* | 03-291010 | KF564752/ SIN065-14 | Pulau Tekong | 1.4143 | 104.0387 |
| 67 | *Cx. (Culex) vishnui* | 04-121110 | KF564733/ SIN066-14 | Jurong West Avenue 3 | 1.3517 | 103.7045 |
| 68 | *Culex (Culex) pseudovishnui* Colless 1957 | 01-200511 | KF564722/ SIN067-14 | EHI laboratory strain | NA | NA |
| 69 | *Cx. (Culex) pseudovishnui* | 02-200511 | KF564723/ SIN068-14 | EHI laboratory strain | NA | NA |
| 70 | *Culex (Culex) tritaeniorhynchus* Giles 1901 | 06-220311 | KF564730/ SIN069-14 | Pulau Tekong | 1.4143 | 104.0387 |
| 71 | *Cx. (Culex) tritaeniorhynchus* | 07-220311 | KF564731/ SIN070-14 | Pulau Tekong | 1.4143 | 104.0387 |
| 72 | *Cx. (Culex) tritaeniorhynchus* | 08-220311 | KF564732/ SIN071-14 | Pulau Tekong | 1.4143 | 104.0387 |
| 73 | *Culex (Culex) quinquefasciatus* Say 1823 | 05-290311 | KF564726/ SIN072-14 | EHI laboratory strain | NA | NA |
| 74 | *Cx. (Culex) quinquefasciatus* | 11-280311 | KF564724/ SIN073-14 | Whampoa Road | 1.3262 | 103.8568 |
| 75 | *Cx. (Culex) quinquefasciatus* | 12-280311 | KF564725/ SIN074-14 | Whampoa Road | 1.3262 | 103.8568 |
| 76 | *Lutzia halifaxii* Theobald 1903 | 01-080714 | KM609458  SIN130-14 | Yishun Avenue 6 | 1.4330 | 103.8453 |
| 77 | *Lutzia fuscana* Wiedemann 1802 | 13-100412 | KF564755/ SIN077-14 | Lim Chu Kang Lane 9 | 1.4455 | 103.7101 |
| 78 | *Lt. fuscana* | 14-100412 | KF564756/ SIN078-14 | EHI laboratory strain | NA | NA |
| 79 | *Culex (Culex) mimulus* Edwards 1915 | 15-230610 | KF564744/ SIN079-14 | EHI laboratory strain | NA | NA |
| 80 | *Cx. (Culex) mimulus* | 16-230610 | KF564745/ SIN080-14 | EHI laboratory strain | NA | NA |
| 81 | *Cx. (Culex) mimulus* | 14-230610 | KF564743/ SIN081-14 | EHI laboratory strain | NA | NA |
| 82 | *Cx. (Culex) mimulus* | 17-230610 | KF564746/ SIN082-14 | Pulau Ubin | 1.4126 | 103.9579 |
| 83 | *Cx. (Culex) mimulus* | 01-141011 | KF564748/ SIN083-14 | Pulau Ubin | 1.4126 | 103.9579 |
| 84 | *Cx. (Culex) mimulus* | 02-140111 | KF564749/ SIN084-14 | Pulau Ubin | 1.4126 | 103.9579 |
| 85 | *Cx. (Culex) mimulus* | 03-140111 | KF564750/ SIN085-14 | Pulau Ubin | 1.4126 | 103.9579 |
| 86 | *Cx. (Culex) mimulus* | 18-230610 | KF564747/ SIN086-14 | Pulau Ubin | 1.4126 | 103.9579 |
| 87 | *Cx. (Culex) mimulus* | 04-140111 | KF564751/ SIN087-14 | Pulau Ubin | 1.4126 | 103.9579 |
| 88 | *Culex (Culex) bitaeniorhynchus* Giles 1901 | 03-220211 | KF564715/ SIN088-14 | Lor Chencharu | 1.4130 | 103.8296 |
| 89 | *Cx. (Culex) bitaeniorhynchus* | 04-220211 | KF564716/ SIN089-14 | Lor Chencharu | 1.4130 | 103.8296 |
| 90 | *Cx. (Culex) bitaeniorhynchus* | 01-220211 | KF564714/ SIN090-14 | Pulau Tekong | 1.4143 | 104.0387 |
| 91 | *Culex (Culex) fuscocephala* (Theobald 1907) | 06-220211 | KF564717/ SIN091-14 | Lim Chu Kang Lane 9 | 1.4455 | 103.7101 |
| 92 | *Cx. (Culex) fuscocephala* | 07-220211 | KF564718/ SIN092-14 | Lim Chu Kang Lane 9 | 1.4455 | 103.7101 |
| 93 | *Cx. (Culex) fuscocephala* | 09-220211 | KF564719/ SIN093-14 | Lim Chu Kang Lane 9 | 1.4455 | 103.7101 |
| 94 | *Culex (Culiciomyia) nigropunctatus* Edwards 1926 | 01-020311 | KF564736/ SIN094-14 | Mandai Lake Road 15 | 1.4074 | 103.7834 |
| 95 | *Cx. (Culiciomyia) nigropunctatus* | 04-020311 | KF564737/ SIN095-14 | Ho Ching Road | 1.3353 | 103.7240 |
| 96 | *Cx. (Culiciomyia) nigropunctatus* | 05-020311 | KF564738/ SIN096-14 | Ho Ching Road | 1.3353 | 103.7240 |
| 97 | *Culex (Culex) gelidus* Theobald 1901 | 07-051211 | KF564753/ SIN097-14 | Pulau Tekong | 1.4143 | 104.0387 |
| 98 | *Cx. (Culex) gelidus* | 06-051211 | KF564720/ SIN098-14 | Pulau Tekong | 1.4143 | 104.0387 |
| 99 | *Cx. (Culex) gelidus* | 08-051211 | KF564721/ SIN099-14 | Sungei Bukit Mandai | 1.4357 | 103.7602 |
| 100 | *Culex (Culex) sitiens* Wiedemann 1828 | 09-230610 | KF564727/ SIN100-14 | Lim Chu Kang Lane 9 | 1.4455 | 103.7101 |
| 101 | *Cx. (Culex) sitiens* | 11-230610 | KF564728/ SIN101-14 | Pulau Ubin | 1.4126 | 103.9579 |
| 102 | *Cx. (Culex) sitiens* | 12-230610 | KF564729/ SIN102-14 | Lim Chu Kang Lane 9 | 1.4455 | 103.7101 |
| 103 | *Culex (Eumelanomyia) brevipalpis* (Giles 1902) | 17-051211 | KF564740/ SIN103-14 | Vigilante Road | 1.2848 | 103.7887 |
| 104 | *Cx. (Eumelanomyia) brevipalpis* | 16-051211 | KF564739/ SIN104-14 | Vigilant Drive | 1.2848 | 103.7887 |
| 105 | *Cx. (Eumelanomyia) brevipalpis* | 27-240412 | KF564754/ SIN105-14 | Vigilant Drive | 1.2848 | 103.7887 |
| 106 | *Aedeomyia (Aedeomyia) catasticta* Knab 1909 | 19-051211 | KF564757/ SIN106-14 | Murai Farmway | 1.3905 | 103.6960 |
| **107** | ***Coquillettidia (Coquillettidia) nigrosignata* (Edwards 1917)** | **12-051211** | **KF564775/ SIN107-14** | **Pulau Tekong** | **1.4143** | **104.0387** |
| **108** | ***Cq.(Coquillettidia) nigrosignata*** | **05-111111** | **KF564773/ SIN108-14** | **Pulau Tekong** | **1.4143** | **104.0387** |
| **109** | ***Cq.(Coquillettidia) nigrosignata*** | **02-200412** | **KF564772/ SIN109-14** | **Pulau Tekong** | **1.4143** | **104.0387** |
| **110** | ***Cq.(Coquillettidia) nigrosignata*** | **06-111111** | **KF564774/ SIN110-14** | **Military training areas** | **NA** | **NA** |
| **111** | ***Coquillettidia (Coquillettidia) crassipes* (Van der Wulp 1881)** | **01-070611** | **KF564770/ SIN111-14** | **Pulau Tekong** | **1.4143** | **104.0387** |
| **112** | ***Cq.(Coquillettidia) crassipes*** | **03-070611** | **KF564771/ SIN112-14** | **Pulau Tekong** | **1.4143** | **104.0387** |
| 113 | *Mansonia (Mansonioides) bonneae* Edwards 1930 | 12-150711 | KF564765/ SIN113-14 | Military training areas | NA | NA |
| 114 | *Mansonia (Mansonioides) uniformis* | 01-091012 | KF564766/ SIN114-14 | Military training areas | NA | NA |
| 115 | *Ma. (Mansonioides) annulata* Leicester 1908 | 04-091012 | KF564764/ SIN115-14 | Pulau Tekong | 1.4143 | 104.0387 |
| **116** | ***Uranotaenia (Uranotaenia) micans* Leicester 1908** | **02-201210** | **KF564767/ SIN116-14** | **Sungei Buloh Wetland Reserve** | **1.4467** | **103.7301** |
| **117** | ***Ur. (Uranotaenia) micans*** | **03-201210** | **KF564768/ SIN117-14** | **Sungei Buloh Wetland Reserve** | **1.4467** | **103.7301** |
| **118** | ***Uranotaenia (Uranotaenia) longirostris* (Leicester 1908)** | **26-051211** | **KF564769/ SIN118-14** | **Sungei Buloh Wetland Reserve** | **1.4467** | **103.7301** |
| 119 | *Ficalbia minima* (Theobald 1901) | 09-111111 | KF564762/ SIN119-14 | Sungei Buloh Wetland Reserve | 1.4467 | 103.7301 |
| 120 | *Fi. minima* | 10-111111 | KF564763/ SIN120-14 | Sungei Buloh Wetland Reserve | 1.4467 | 103.7301 |
| 121 | *Fi. minima* | 08-111111 | KF564761/ SIN121-14 | Sungei Buloh Wetland Reserve | 1.4467 | 103.7301 |
| 122 | *Armigeres (Armigeres) subalbatus* (Coquillett 1898) | 04-151111 | KF564760/ SIN122-14 | Murai Farmway | 1.3905 | 103.6960 |
| 123 | *Armigeres (Armigeres) subalbatus* | 02-151111 | KF564758/ SIN123-14 | Pulau Ubin | 1.4126 | 103.9579 |
| 124 | *Ar. (Armigeres) subalbatus* | 03-151111 | KF564759/ SIN124-14 | Pulau Ubin | 1.4126 | 103.9579 |
| **125** | ***Armigeres (Armigeres) kesseli* Ramalingam 1987** | **34-240412** | **KF564712/ SIN125-14** | **Military training areas** | **NA** | **NA** |
| **126** | ***Zeugnomyia gracilis* Leicester 1908** | **18-130112** | **KF564776/ SIN126-14** | **Dairy Farm Road** | **1.3647** | **103.7733** |
| **127** | ***Ze. gracilis*** | **33-240412** | **KF564778/ SIN127-14** | **MacRitchie Reservoir** | **1.3475** | **103.8137** |
| 128 | *Toxorhynchites (Toxorhynchites) splendens* (Wiedemann 1819) | 12-130112 | KF564777/ SIN128-14 | Telok Blangah Green | 1.2789 | 103.8133 |

**^§^***COI* gene sequences of mosquito species (n=16) reported for the first time in the present study are highlighted in bold letters. *COI* gene sequences of those species were not available previously in public sequence databases.
